# SOCIAL ISOLATION IN OLDER ADULTS POST HIP SURGERY IS CORRELATED WITH MOBILITY AND PHYSIOLOGICAL INDICATORS

**DOI:** 10.1093/geroni/igad104.2821

**Published:** 2023-12-21

**Authors:** Faranak Dayyani, Charlene Chu, Ali Abedi, Shehroz Khan

**Affiliations:** University of Toronto, Toronto, Ontario, Canada; University of Toronto, Toronto, Ontario, Canada; University Heatlh Network, Toronto, Ontario, Canada; Toronto Rehabilitation Institute, Toronto, Ontario, Canada

## Abstract

Hip fracture affect approximately 30,000 people in Canada and more than 300,000 people in U.S., annually. Older adults post hip-fracture surgery experience social isolation along with reduced physical mobility, changes in sleeping patterns, reduced indoor and outdoor activities, and other physiological changes once they are discharged form inpatient rehabilitation. Our team has developed MAISON (Multimodal AI-based Sensor platform for Older iNdividuals), a cloud-based multimodal sensor system that supports the collection of physiological, ambient and contextual data from various smart devices. Currently, MAISON consists of a smart watch, a smart phone, a motion sensor and a sleep mattress. Using MAISON, we have collected 24 weeks of raw acceleration data, heartrate, step count, frequency of indoor motion, GPS and sleep metrics from three older adults post-hip surgery living in the community. Statistical and domain-specific features were extracted from the collected data with a time window of one day, including average heartrate, maximum acceleration, total sleep time, total steps taken, and average number of exits. Additionally, clinical data is collected from the participants on a biweekly basis consisting of three questionnaires (i.e., Social Isolation Scale (SIS)) and two physical tests. The correlation between features from sensor data and clinical data was performed using Spearman coefficient, which showed a strong positive correlation (>0.5) between the SIS and number of exits, variance of acceleration, total sleep time, and heartrate variance. Various correlation values were found between features from all the sensors and SIS, indicating the usefulness of multimodal sensors for this application.

